# Application of enhanced recovery after surgery care protocol in the perioperative care of patients undergoing lumbar fusion and internal fixation

**DOI:** 10.1186/s13018-022-03099-0

**Published:** 2022-04-18

**Authors:** Zengmei Sun, Yanqiu Qi

**Affiliations:** 1 Weifang People’s Hospital, Weifang, 261041 Shandong China; 2 Department of Neck and Lumbar Pain, and Bone Injuries Rehabilitation, Weifang Hospital of Traditional Chinese Medicine, No. 1055, Weizhou Road, Kuiwen District, Weifang, 261041 Shandong China

**Keywords:** Lumbar fusion and internal fixation, Enhanced recovery after surgery, Perioperative care

## Abstract

**Background:**

To explore the effects and deficiencies of the enhanced recovery after surgery (ERAS) care protocol on patients undergoing lumbar fusion and internal fixation in perioperative care.

**Methods:**

A total of 166 patients with lumbar fusion and internal fixation were collected and divided into two groups, among which 86 patients received ERAS care protocol were attributed into ERAS group, while the other 80 patients received traditional perioperative care protocol were assigned to control group. Then, the degree of pain, self-care ability and the degree of recovery were assessed using the visual analogue scale (VAS), Barthel index (BI) rating scale and the Sino-version Oswestry Disability Index (ODI) questionnaire, respectively. Moreover, further analysis was performed based on patients’ different age, gender, body mass index (BMI) and education of patients in ERAS group.

**Results:**

The hospitalization time and the incidence of complication in the ERAS group were obviously lower than those in control group (both, *P* < 0.05). There was no significant difference in hospitalization expenses between the two groups (*P* > 0.05). The BI score of the ERAS group was higher than that of the control group (*P* < *0.05*), and the percentage of ODI score in ERAS group was significantly downregulated in comparison with that in control group (*P* < *0.05*). Intra-group analysis in ERAS showed that, compared with older patients, younger patients had higher BI scores (*P* < 0.05) and lower ODI scores (*P* < 0.05); meanwhile, overweight patients had lower BI scores (*P* < 0.05), and the ODI score decreased with the increase in education level of the patients.

**Conclusions:**

ERAS care protocol can significantly shorten the hospitalization time and reduce the occurrence of postoperative complications of patients, significantly enhance the self-care ability of patients after discharge and promote the rapid recovery of patients after surgery.

**Supplementary Information:**

The online version contains supplementary material available at 10.1186/s13018-022-03099-0.

## Introduction

Lumbar degenerative disease is a common disease in the elderly, which is characterized by lumbar and leg pain and motor dysfunction and seriously affects the quality of patients’ life [1, 2]. Surgery is the main clinical treatment for patients with severe lumbar degenerative diseases [3], among which spinal fusion and internal fixation is one of the most important methods of treatment [4]. However, due to the long operation time of lumbar fusion internal fixation, the great trauma to the patients and the long postoperative bed rest of the patients, the postoperative recovery speed of the patients is delayed to a large extent; moreover, the physical and psychological burden of the patients is increased [5]. Therefore, it is urgent to explore a more optimized perioperative nursing plan to promote postoperative rehabilitation of patients.

Enhanced recovery after surgery (ERAS) refers to the adoption of a variety of effective treatment measures to reduce a variety of physiological and psychological adverse reactions caused by surgery, so as to promote the rapid recovery of patients [6, 7]. And the proposition of ERAS has opened up a new way of thinking to solve the problem of rapid recovery of patients after various operations and to save the cost of treatment [8]. Actually, early clinical studies of ERAS have been carried out in European and American countries. Briefly, ERAS is first used in colorectal surgery and later in other surgical fields such as urology, breast surgery and gynecology [8]. More importantly, the efficacy of ERAS in patients undergoing spinal surgery has also been demonstrated during the past decade. For example, Smith et al. reports that implementing an ERAS bundle for 1–2-level lumbar fusion has minimal effect in decreasing length of stay, but a significant decrease in postoperative opioid and rescue antiemetic use [9]. Debonoet et al. have explored the influences of ERAS nursing plan on the postoperative complications, pain and the length of hospital stay in the patient who underwent spinal fusion and internal fixation and found that ERAS is suitable to spinal surgery [10]. However, the development of ERAS in China is still in the infancy and there is a lack of clinical data support from different nursing specialties.

In current study, we first analyzed and compared the differences in length of stay, cost of stay, postoperative complications, degree of pain, self-care ability and degree of recovery between patients using ERAS nursing plan and traditional nursing plan during perioperative period. This study will provide a powerful theoretical support for the application and effect of ERAS nursing program in nursing work, as well as a reference for rehabilitation nursing of patients undergoing lumbar fusion and internal fixation.

## Materials and methods

### Patients and grouping

A total of 166 patients who underwent lumbar fusion and internal fixation in the spine surgery of Weifang Medical University from January 2019 to April 2021 were collected in our study. Moreover, the study was approved by the Ethics Committee of Weifang People's Hospital, and informed consent was obtained from each participant. All the patients were divided into two groups: 80 patients receiving traditional nursing were enrolled in the control group, and another 86 patients receiving ERAS nursing plan at perioperative period were enrolled in ERAS group.

### Eligibility criteria

Participants who meet the criteria below were included: (1) The patient was clinically diagnosed with lumbar spinal stenosis, spondylolisthesis or lumbar disk herniation; (2) lumbar fusion and internal fixation were planned for the treatment. Meanwhile, the exclusion criteria were as follows: (1) The patients were diagnosed with a spinal infection or tumor when they were at hospital; (2) patients with severe heart, lung, liver, kidney and other organ function damage and metabolic dysfunction; (3) patients with a disease of the blood system, such as coagulation dysfunction; (4) patients with severe mental illness and cognitive impairment; (5) patients with a history of lumbar surgery; (6) patients with prolonged hospitalization due to other reasons; (7) patients who could not carry out rehabilitation exercise according to nursing requirements; and (8) patients with incomplete clinical data and postoperative follow-up data.

### Nursing intervention

For patients in control group, traditional nursing plan was conducted, including admission assessment, surgical education, preoperative preparation, postoperative nursing and discharge guidance. With respect to patients in experiment group, the ERAS nursing plan was conducted, which mainly contained educational program, management of nutrition, management of dietary, management of sleep, management of pain, management of body temperature, liquid therapy, postoperative diet, postoperative functional exercise, pipeline care and get out of bed early after surgery. The detailed information for traditional care and ERAS nursing plan is shown in Additional file 1: Table S1.

### Evaluation index

The number of patients with postoperative complications, such as delayed wound healing, poor wound healing and urinary system infection, was observed and recorded. The criteria were as follows: (1) delayed wound healing. Usually, the suture removal can be completed in 10–12 days after the operation of the lower back; hence, delayed wound healing can be diagnosed if the patient’s wound does not fully heal within 10 to 12 days after lumbar spine surgery; (2) poor wound healing: generally refers to the healing of red swelling, induration, effusion, purulent and other inflammatory reactions; and (3) urinary system infection: The diagnosis of urinary system infection was made by the urologists according to the diagnostic criteria of urinary system infection in the “Diagnostic criteria for hospital infection (trial)” issued by the Ministry of Health in 2001.

Moreover, patients' pain was assessed using a visual analogue scale (VAS) 1 month after discharge. Briefly, VAS = 0 means “no pain” (score 0) and VAS = 10 means “pain as bad as it could be”; 0 < VAS ≤ 3, the pain is mild and tolerable; 4 ≤ VAS ≤ 6, the pain is more pronounced and represents moderate pain; and 7 ≤ VSA ≤ 10, the pain is very intense and intolerable and represents severe pain.

Barthel index (BI) rating scale [11], the most widely used personal self-care ability assessment scale with good reliability and validity in the world, was used to evaluate the self-care ability of the patients 1 month after discharge, which included patients' eating, bathing, grooming, dressing, stool control, urine control, toilet use, bedchair transfer, walking 45 cm on the ground and walking up and down stairs. On a scale of 100, the higher score indicated better independence and less dependence [12]. Briefly, a score of 40 or less was defined as heavy dependence; a score of 41 to 60 was defined as moderate dependence; a score of 61 to 99 was defined as mild dependence; and a score of 100 is defined as no dependency.

Oswestry Disability Index (ODI) questionnaire was used to assess the extent of the patient's recovery after 1 month of discharge. ODI has been the gold standard for assessment of lumbar function [13, 14], which contained the degree of pain, daily life self-care (washing, clothes and other activities), lifting, walking, sitting, standing, sleeping, social activities and travel (outing). Each item has a maximum score of 5, with a higher total score indicating more severe dysfunction.

### Intra-group analysis of patients receiving ERAS care

In order to perform an intra-group analysis of patients receiving ERAS care, 86 patients in experimental group were further distributed to different groups. Briefly, based on median age (57 years), the patients were divided into younger and older patients groups, respectively. Then, the patients in the experiment group were also divided into underweight (BMI < 18.5 kg/m^2^), normal weight (BMI = 18.5–23.9 kg/m^2^) and overweight (BMI ≥ 24 kg/m^2^) groups, respectively. Moreover, based on the level of education, patients were attributed to primary school (9 cases), junior middle school (28 cases), technical secondary school and senior high school (35 cases) and junior college or above (14 cases) groups.

### Statistical analysis

The data were analyzed using SPSS 21.0 software and presented as mean ± SD and GraphPad Prism 7.0 software. The difference between two groups and among three or more groups was compared by Student’s t test and Chi-square test, respectively. *P* < 0.05 meant the difference was significant.

## Results

### The baseline data of patients in two groups

As shown in Table 1, the clinical data of patients in two groups were analyzed. The results demonstrated that there was no significant difference in the age, gender, BMI, the level of education, preoperative diagnosis, preoperative VAS score, the time of operation and perioperative bleeding of patients between ERAS group and control group (*P* > 0.05), indicating that the general data of the two groups were comparable.

**Table 1:** The baseline of patients in two group (${\overline{\text{x}}} \pm {\text{s}}$)

Indexes	Control group (*n* = 80)	Experiment group (*n* = 86)	*t*/*χ*^*2*^	*P* value
Age	58.863 ± 10.880	56.919 ± 11.699	1.106	0.270
Gender (male/female)	35/45	27/59	2.704	0.100
BMI (kg/m^2^)	24.411 ± 2.641	24.821 ± 2.593	− 1.009	0.315
The level of education			0.441	0.660
Primary school	9	9		
Junior high school	12	28		
Technical secondary school and high school	25	35		
University or college education	34	14		
Preoperative diagnosis			0.024	0.988
Spinal canal stenosis	56	61		
Lumbar spondylolisthesis	18	19		
Lumbar disk herniation	6	6		
Preoperative VAS score	5.175 ± 2.238	5.023 ± 2.608	0.401	0.689
The time of operation (min)	94.675 ± 17.298	92.919 ± 16.013	0.679	0.498
Perioperative bleeding (mL)	193.529 ± 58.913	198.666 ± 59.710	− 0.557	0.578

*BMI* Body mass index, *VAS* visual analogue scale

### Comparison of observation indexes during hospitalization

The observation indexes during hospitalization including the hospital stays, hospitalization cost and postoperative complications of patients were explored. As a result, the hospital stays and hospitalization cost were both lower in ERAS group than those in control group, while only hospital stays were significantly different between two groups (*P* = 0.001; Table 2). Moreover, the complication rate in ERAS group (5.81%) was obviously lower than that in control group (16.25%) (*P* = 0.044; Table 2). Specially, delayed wound healing was the most common type of complication in both groups.

**Table 2:** The observation indexes between the two groups during hospitalization (${\overline{\text{x}}} \pm {\text{s}}$)

	Control group (*n* = 80)	Experimental group (*n* = 86)	*t*/*χ*^*2*^	*P* value
Hospital stays (d)	12.050 ± 3.467	10.465 ± 2.237	3.524	0.001
Hospitalization cost (ten thousand yuan)	3.746 ± 0.712	3.547 ± 0.746	1.756	0.081
Postoperative complications	13 (16.25%)	5 (5.81%)	–	0.044
Delayed wound healing	7	3		
Poor healing of the cutting edge	4	1		
Urinary system infection	2	1		

### Observation index comparison of patients after discharge

**Fig. 1:**
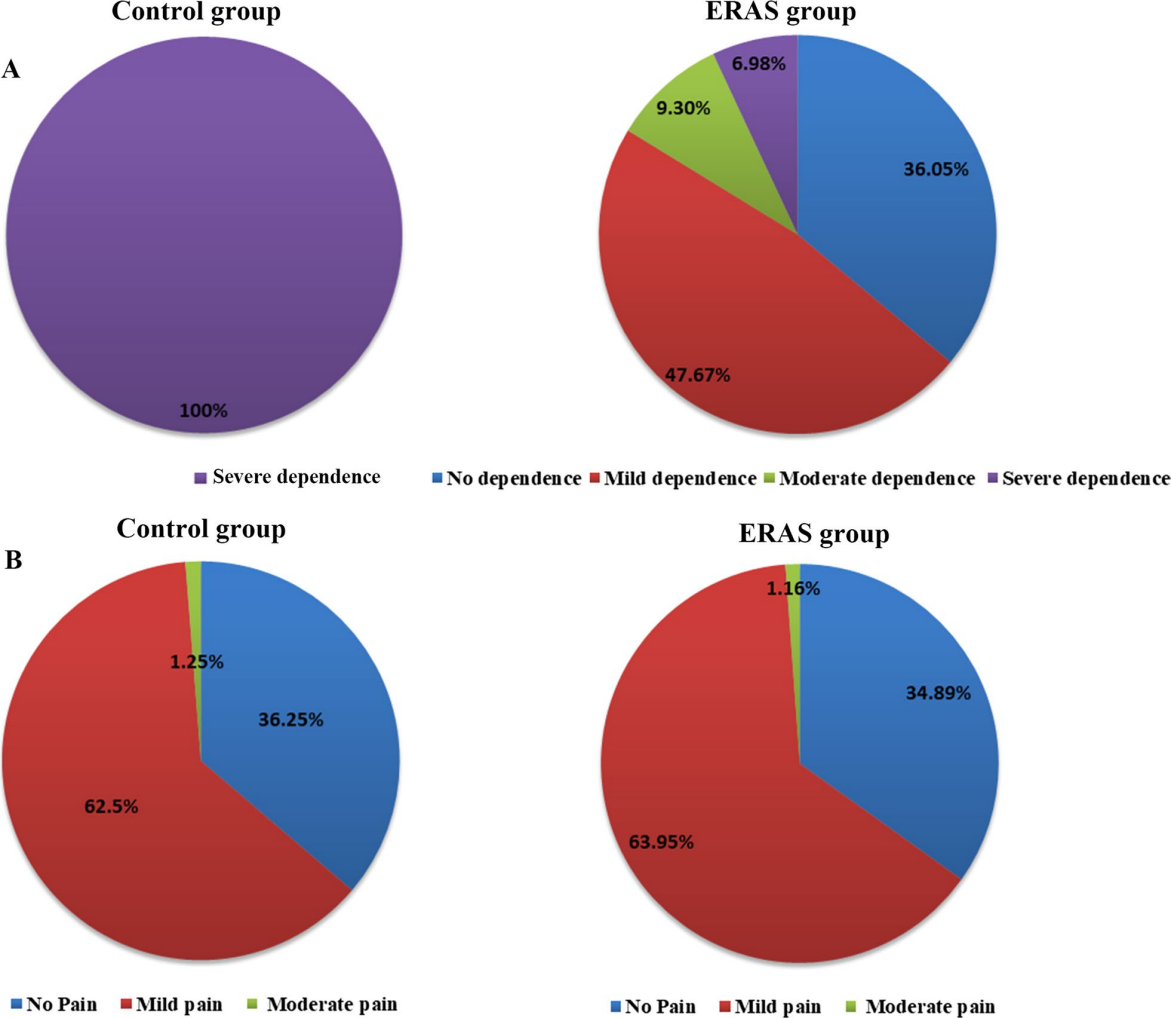
Observation index comparison of patients after discharge. **A** The self-care ability of patients in ERAS group and control group. **B** Distribution of pain of patients in ERAS group and control group

To investigate the condition after discharge of patients, a follow-up phone call was conducted for all patients and the value of VAS, BI and ODI was recorded and compared. The results demonstrated that the BI value in ERAS group was significantly increased in comparison with that in control group (*P* < 0.05, Table 3), suggesting the self-care ability of patients was significantly improved after ERAS care protocol in perioperative care. Moreover, all the patients in control group were severely dependent, while the dependent degree of patients in ERAS group was significantly improved, with only 6 (6.98%) patients with severe dependence, 31 (36.05%) patients with no dependence, 41 (47.67%) patients with mild dependence and 8 (9.30%) patients with moderate dependence (Fig. 1A).

**Table 3:** The comparison of observation index of patients in two groups after discharge one month (${\overline{\text{x}}} \pm {\text{s}}$)

	Control group	Experimental group	*t*	*P*
VAS score	1.263 ± 1.156	1.081 ± 1.031	1.067	0.288
BI score	21.400 ± 11.208	81.047 ± 24.479	− 19.934	< 0.001
ODI score (%)	78.219 ± 3.540	25.276 ± 50.841	9.291	< 0.001

*VAS* Visual analogue scale, *BI* Barthel index, *ODI* Oswestry Disability Index

The ODI value of patients in ERAS group was lower than in control group, which indicated that ERAS care protocol in the perioperative care could enhance the extent of the patient's recovery.

Furthermore, the VAS of patients in ERAS group was reduced but not significantly different from that in control group (*P* = 0.288 > 0.05, Table 3). Meanwhile, in ERAS group, 30 patients (34.89%) had no pain, 55 patients (63.95%) had mild pain, and 1 patient (1.16%) had moderate pain. In control group, there were 29 (36.25%) patients without pain, 50 (62.50%) patients with mild pain, and 1 (1.25%) patients with moderate pain (Fig. 1B).

### Intra-group analysis of general data of patients in ERAS group

To analyze the deficiencies of ERAS within the currently used care programs, an in-depth analysis based on general clinical data related to nursing outcomes in ERAS group was conducted. Firstly, all patients in ERAS group were divided into different subgroups. As illustrated in Table 4, the BI score was higher, while the ODI score was lower in younger patients group than that in older patients group (*P* < 0.05). Moreover, there was no significant difference between female and males groups in the hospital stays, hospitalization cost and postoperative complications, VAS score, BI score and ODI score (*P* > 0.05). What’s more, the VAS score and ODI score in patients with normal BMI were both lower than those in patients with overweight (*P* < 0.05). Besides, the ODI score was notably reduced along with the increase in education level of patients (*P* < 0.05).

**Table 4:** Analysis results based on general data of patients in the trial group (${\overline{\text{x}}} \pm {\text{s}}$)

Groups	Age		Gender		BMI					Education level	
	Low age	Advanced age	Male	Female	Flat	Normal	Overweight	Primary school	Junior high school	Technical secondary school and high school	College degree or above
Number	44	42	27	59	1	33	52	9	28	35	14
Hospital stays (day)	10.59 ± 2.48	10.33 ± 1.97	10.37 ± 2.42	10.51 ± 2.17	12.00	10.30 ± 2.28	10.54 ± 2.24	10.11 ± 2.37	10.64 ± 2.38	10.46 ± 2.31	10.36 ± 1.87
Hospitalization cost (ten thousand yuan)	3.66 ± 0.73	3.43 ± 0.75	3.72 ± 0.52	3.47 ± 0.82	3.00	3.63 ± 0.53	3.51 ± 0.86	3.72 ± 1.08	3.52 ± 0.69	3.50 ± 0.69	3.63 ± 0.81
Postoperative complications	1	4	2	3	0	0	5	0	3	2	0
VAS score	1.09 ± 0.96	1.071 ± 1.11	1.07 ± 0.96	1.09 ± 1.07	0	0.49 ± 0.67	1.48 ± 1.03^b^	0.93 ± 0.71	1.15 ± 1.22	1.10 ± 0.85	0.99 ± 0.10
BI score	87.98 ± 17.38	73.79 ± 28.63^a^	79.63 ± 26.90	81.70 ± 23.50	100	79.85 ± 28.18	81.44 ± 22.19	69.43 ± 18.49	74.86 ± 34.35	88.37 ± 30.26	92.55 ± 44.15
ODI score (%)	14.68 ± 19.74	36.37 ± 68.58^a^	22.41 ± 24.47	26.59 ± 59.28	0	6.90 ± 11.90	37.42 ± 61.95^b^	34.86 ± 84.35	24.37 ± 19.26	18.43 ± 24.49	9.55 ± 14.15^c^

*VAS* Visual analogue scale, *BI* Barthel index, *ODI* Oswestry Disability Index

^a^VS younger *P* < 0.05; ^b^VS normal *P* < 0.05; ^c^VS educational level *P* < 0.05

## Discussion

As a concept that optimizes and integrates perioperative treatment and care, ERAS is widely used in a variety of surgical specialties around the world [15]. Although ERAS has been commonly used in other musculoskeletal procedures, such as total joint replacement, its use in spine surgery has been slow to develop and researches on this concept have been limited [16], especially in China. Hence, in this study, the application effect of ERAS nursing plan and traditional nursing plan in patients undergoing lumbar fusion internal fixation was compared, and the findings will provide clinical theoretical support for the application of ERAS nursing plan in spine surgery.

Previous study has report that the length of hospital stay in the ERAS cohort was significantly shorter than that in the control cohort [17]. The consequences of the present study showed that the hospital stays of patients in ERAS group were significantly shorter than that in control group, which might benefit from a well-planned preoperative examination, early postoperative diet and early postoperative functional exercise. Hospitalization cost has always been a concern of patients. Usually, hospitalization costs are usually directly or indirectly related to gender, age, urban and rural distribution and education level of patients [18]. In this study, we found that the hospitalization cost of patients in ERAS group was slightly less than that in control group; however, the difference was not significant.

Patients underwent spinal fusion often suffer from a variety of complications, among which postoperative incisional complications are the main complications after various types of surgery [18]. Usually, incisional complications are not life-threatening, but can reduce the quality of life of patients and increase the burden of medical care costs [19]. It has been reported that perioperative continuous hypothermia will cause adverse cardiovascular events, reduce the immune function of the body and cause coagulation dysfunction, thus affecting the wound healing [20]. In this study, there was a statistically significant difference in the total incidence of complications between ERAS and control groups, suggesting that the ERAS care protocol may reduce the incidence of postoperative complications after lumbar fusion internal fixation. The largest percentage of postoperative complications in both groups was delayed wound healing, while pressure ulcers, venous thrombosis and pulmonary infections did not occur. Additionally, the complications of the incision in this study included delayed wound healing and poor wound healing. Briefly, a total of 13 patients (16.25%) developed postoperative complications, including 11 incisional complications (delayed wound healing and poor wound healing) and 2 urinary infections in control group, while only 5 patients (5.81%) had complications, including 4 incisional complications and 1 urinary tract infection in ERAS group. All these findings suggested the implementation of ERAS nursing program is very helpful to reduce the incidence of postoperative complications. Therefore, the application of ERAS in perioperative nursing of lumbar fusion and internal fixation should focus on the occurrence of delayed wound healing and seek effective methods to reduce the incidence of this complication.

Pain is the fifth most important vital sign after body temperature, pulse, respiration and blood pressure, which is the main cause of medical treatment for most patients in spinal surgery. According to the study of Vilmarsson et al., painful stimulation will cause sympathetic nerve reflex and blood vessel and muscle contraction after surgery, which will lead to insufficient blood supply to the surgical incision, eventually delaying wound healing and increasing the chance of infection [21]. Hence, pain relief is especially important in preventing infection in patients undergoing lumbar surgery. The data in our study indicated that the VAS score in ERAS group was lower than that of patients in control group, but the difference did not reach a statistical level, indicating that the current ERAS nursing plan was very helpful for the reduction of pain in ERAS group, but there were still shortcomings. Moreover, further analysis in ERAS group of patients showed that the VAS score of overweight patients was significantly higher than that of normal BMI patient, indicating overweight is a major risk factor for pain, especially in patients undergoing spinal surgery.

The functional disorder of lumbar vertebra will seriously affect the movement and self-care ability of patients once pathological changes occur, which will significantly reduce the quality of life of patients [22]. BI score is now widely used in the evaluation of patients with self-care ability of the key indicators [11]. In this study, we found that the BI value in ERAS group was significantly increased in comparison with that in control group. This suggested that ERAS significantly improves the ability of patients to take care of themselves one month after discharge and facilitates the recovery process. Next, further analysis of in ERAS group revealed that the BI score of self-care ability and dependence of elderly patients in ERAS group were significantly lower than those of younger patients. Actually, Musa et al. have proved that age was an important factor affecting the change of BI score [23]. Hence, we speculate that it is associated with the lower average physical quality, the lower body resistance and the lower metabolic activity of the elderly.

For patients undergoing lumbar spine surgery, the ODI score is most commonly used to assess outcome and is the “Gold standard” for evaluation [24]. The present study found that the ODI score of the control group was significantly higher than that of the ERAS group, which indicated that ERAS nursing program can significantly promote the progress of rehabilitation of patients undergoing lumbar spinal fusion and internal fixation. Previous study proved that dysfunction was age-dependent, and younger patients often showed better postoperative improvement after surgery than older patients [25]. Besides, BMI and the educational level are the most important impact indicators of postoperative functional rehabilitation [26, 27]. However, the data in this study demonstrated that there was no progress in the degree of rehabilitation of patients with advanced age, overweight and low education levels.

In conclusion, this study revealed that ERAS nursing plan can significantly shorten the hospital stay of patients undergoing lumbar fusion internal fixation, reduce postoperative complications, improve self-care ability after discharge and promote rapid postoperative recovery of patients. Moreover, ERAS had no significant effect on the cost of hospitalization and postoperative pain, suggesting that the future nursing work should focus on improving and developing effective measures to reduce the financial burden of patients and improve their quality of life.

## Supplementary Information

**Additional file 1: Table S1**. The nursing plan of patients in control group and experiment group.

## Data Availability

Corresponding authors may provide data and materials for this study upon reasonable request.
