# Supplementary material for: Application of enhanced recovery after surgery care protocol in the perioperative care of patients undergoing lumbar fusion and internal fixation
Source: J Orthop Surg Res. 2022 Apr 18;17:240. doi: 10.1186/s13018-022-03099-0 (PMC9014593; doi:10.1186/s13018-022-03099-0)
Supplement: Supplementary file 1 — Additional file 1: Table S1. The nursing plan of patients in control group and experiment group. [file 13018_2022_3099_MOESM1_ESM.docx]

Supplementary table 1. The nursing plan of patients in control group and experiment group

|  | ERAS nursing plan | Traditional nursing plan |
| --- | --- | --- |
| Admission assessment | Routine admission introduction was conducted and the physical and mental condition were estimated. Moreover, the medical history, past history, allergy history, smoking and drinking history, medication history, and specialized symptoms and signs of patients were asked in detail. Meanwhile, the Body Mass Index (BMI) was calculated to understand the patient's urine, feces and skin conditions. The indexes such as pressure ulcers, falls, venous thrombus embolism (VTE), pain degree and self-care ability were used to complete the related estimation. Furthermore, various risk precautions and preventive measures were publicized and instructed; meanwhile, the warning labels of the corresponding high-risk assessment items were hoisted. | Based on routine admission assessment, the nutritional status of the patient was also required to be monitored in experiment group; moreover, the patient's psychological status was taken as the focus of the work content, which need to be known through the way of inquiry or conversation so that the psychological intervention could be done in advance. |
| Surgical education and preoperative preparation | Traditional surgical education including give up smoking and alcohol since admission, and the basic knowledge of the spinal diseases, the correct way to wear the waist, and stay in bed and axis turn was carried out for patients in control group. At the same time, the appropriate preoperative preparations were made as below: 1) bowel preparation: mechanical bowel preparation was generally not done in the department of spine orthopedics and the time of food and drink abstinence was 12 hours before surgery and 8 hours before surgery; 2) skin preparation of operation site: scrub the back of the patients with soap before lumbar surgery; 3) other preoperative precautions: the nurse should tell the patients the methods of surgical and anesthetic, teach them to train their defecation and deep breathing exercises in bed and assist them to prepare urinal pot, measuring cup and other postoperative necessities. | In addition to the content of the traditional surgery mission, ERAS nursing plan more emphasis on the patient's psychological nursing, including emphatically introduce ERAS related content, the diagnosis and treatment methods of spinal disorders, anesthesia, surgical procedure, postoperative likely scenario and response, general discharge time via the application of card, materials, boards and books, to alleviate patients' nervousness, anxiety and fear and patients could know the important role they would in the process of diagnosis and treatment, mobilize the enthusiasm of patients, and obtain the understanding and cooperation of their families through this way. Moreover, the patients were informed of the approximate duration of the operation, the possible situation and intervention measures after the operation, as well as the general time of discharge after the operation telling the successful cases. What’s more, let the patient know that the responsible nurse was experienced and would work side by side with the patients to solve the difficulties of the patients in a timely manner and make the patients feel relaxed. Besides, the nurse should teach the patients how to gradually get out of bed under the protection of the waist, instruct patients to perform functional exercises such as straight leg raising and ankle pump after surgery, conduct health education on pain, including the cause, time and analgesic methods of perioperative pain. As respect to bowel preparation, on the basis of the intestinal preparation of the traditional nursing plan, the food and drink restriction time of the patients was adjusted to preoperative fasting for 6 hours and drinking restriction for 2 hours. The patients could drink sugar water and clear water 2 hours before the surgery and eat starch solid food 6 hours before the surgery, but the fried food, fat and meat food required a longer fasting time, which could not only increase the patient's physical reserve to deal with the trauma of surgery, but also reduce the insulin resistance after surgery. In addition to the traditional preoperative precautions, patients were required to prepare pure cotton sheets and turn over pads and other postoperative necessities. ERAS care protocol increased the preoperative attention to the patient's sleep condition, and sleeping drugs should be given according to the doctor's advice for patients with sleep difficulties so that patients can get adequate rest before surgery. |
| Postoperative care | Routine postoperative handover and postoperative education were conducted and the rapid fluid replacement treatment should be given if the patient's condition permits. Then postoperative pain and gastrointestinal function were assessed and told the patient to try to drink a moderate amount of warm water in 6 hours after the operation, and a liquid and digestible diet was given to the patients if there was no discomfort and slowly returned to normal diet. Moreover, the nurse should guide the patients to do flexion and extension exercises and deep breathing exercises on the bed, and instruct and assist the patient axis to turn. The routine piping care was also performed | On the basis of traditional postoperative nursing measures, the ERAS nursing plan should also do the following. The [body](file:///E:\Program%20Files\Youdao\Dict\7.2.0.0703\resultui\dict\?keyword=body) [temperature](file:///E:\Program%20Files\Youdao\Dict\7.2.0.0703\resultui\dict\?keyword=temperature) of the patients must be kept below 36 degrees and the details of liquid treatment were improved. Besides, pain care was added and the patient's pain and gastrointestinal function should be evaluated systematically and in real time, and timely treatment measures should be given. It was different from the traditional 6 hours after the operation into the diet, patients with ERAS plan advocated early postoperative diet and water or slag-free beverages when fully awake. In addition to flexion and extension exercises, the patient's postoperative functional exercises in bed were mainly adjusted to straight leg raising and ankle pump exercises and the patients could participant in formulating the exercise plan. What’s more, the ERAS care program focused on the early removal of drainage tubes and catheters and corresponding care ERAS. Besides, the ERAS nursing plan required patients to get out of bed early as instructed by the doctor, and the responsible nurse guides and assisted the patients to wear braces and guided the patients to get out of bed gradually, accompanied by their family members during the whole process ERAS care program. |
| Discharge instruction | After the patient in control group recovered, the nurse should continue to guide their functional exercise and bed time, and give regular visits at any time. | In addition to routine discharge guidance, ERAS care program assisted the doctor in charge to evaluate discharge indicators, and make the doctor work with patients to develop discharge diet plan and functional exercise plan. The patients should leave the contact information of the attending physician and the responsible nurse behind so that they can consult at any time and give professional answers. |
